# Effects of Age and Task Load on Drivers’ Response Accuracy and Reaction Time When Responding to Traffic Lights

**DOI:** 10.3389/fnagi.2016.00169

**Published:** 2016-07-12

**Authors:** Emilie Salvia, Claire Petit, Stéphane Champely, René Chomette, Franck Di Rienzo, Christian Collet

**Affiliations:** ^1^Laboratoire de Neurosciences Cognitives, UMR 7291, Centre National de la Recherche Scientifique and Aix-Marseille UniversitéMarseille, France; ^2^Renault TechnocentreGuyancourt, France; ^3^Laboratoire sur les Vulnérabilités et l’Innovation dans le Sport, Université Claude Bernard Lyon 1Villeurbanne, France; ^4^Ecole de Conduite Française – CESRBron, France; ^5^Inter-University Laboratory of Human Movement Biology, Université Claude Bernard Lyon 1Villeurbanne, France

**Keywords:** reaction time, response accuracy, driving, aging, cognitive impairment

## Abstract

Due to population aging, elderly drivers represent an increasing proportion of car drivers. Yet, how aging alters sensorimotor functions and impacts driving safety remains poorly understood. This paper aimed at assessing to which extent elderly drivers are sensitive to various task loads and how this affects the reaction time (RT) in a driving context. Old and middle-aged people completed RT tasks which reproduced cognitive demands encountered while driving. Participants had to detect and respond to traffic lights or traffic light arrows as quickly as possible, under three experimental conditions of incremental difficulty. In both groups, we hypothesized that decision-making would be impacted by the number of cues to be processed. The first test was a simple measure of RT. The second and third tests were choice RT tasks requiring the processing of 3 and 5 cues, respectively. Responses were collected within a 2 s time-window. Otherwise, the trial was considered a no-response. In both groups, the data revealed that RT, error rate (incorrect answers), and no-response rate increased along with task difficulty. However, the middle-aged group outperformed the elderly group. The RT difference between the two groups increased drastically along with task difficulty. In the third test, the rate of no-response suggested that elderly drivers needed more than 2 s to process complex information and respond accurately. Both prolonged RT and increased no-response rate, especially for difficult tasks, might attest an impairment of cognitive abilities in relation to aging. Accordingly, casual driving conditions for young drivers may be particularly complex and stressful for elderly people who should thus be informed about the effects of normal aging upon driving.

## Introduction

Driving requires processing large amounts of information simultaneously, e.g., external information about other drivers, road signs, traffic lights, in-vehicle information and individual information related to one’s own driving actions. Management of such large amounts of information requires to select useful cues and to give priority to the most relevant. Overall, a given set of information elicits mental load, which is determined by the task’s complexity, as well as the drivers’ skills and their own experience of driving. Stress elicited by task characteristics interacts with intrinsic factors, notably the individual perception of difficulty. This subjective perception refers to the strain concept (Gaillard, 1993). The perceived difficulty depends on drivers’ skills and is mediated by anxiety state (Luczak and Göbel, 2000). Hence, stress and strain both contribute to increase mental load. This interaction can lead to overloaded situations and have detrimental effects on performance, particularly on reaction time (RT), response accuracy or both (Ninio and Kahneman, 1974). Accordingly, attentional resources required to process information are mediated by both task difficulty and its subjective perception (Kantowitz, 1987).

In most western countries, 12 to 15% of drivers are over the age of 65, and this segment of the driving population is growing faster than any other (Cantin et al., 2009). Anstey et al. (2005), Cassavaugh and Kramer (2009), and Ferreira et al. (2012) reported that elderly drivers experience more strain than younger drivers due to the changes in cognitive abilities that accompany aging. Therefore, the mental workload experienced by elderly drivers may be harder to manage (Cantin et al., 2009). The elderly often process information more slowly than younger individuals, and the time allocated to process information is often incompatible with driving demands (Warshawsky-Livne and Shinar, 2002; Cantin et al., 2009). Individual information-processing abilities may become overwhelmed (Boucsein and Backs 2000, 2009) and this can impair driving safety, especially under high temporal constraints. Moreover, since elderly drivers are aware of their impaired abilities, difficult driving conditions can increase their anxiety level and concomitantly reduce their ability to process information efficiently. Relevant information can thus be omitted due to excessive strain (Gaillard and Kramer, 2000). Most studies reported that elderly drivers avoid complex driving situations in which they do not feel confident, e.g., night-time driving, traffic-jams, bad weather, or even when they know that they will have to perform complex maneuvers (Baldock et al., 2006; Charlton et al., 2006; Blanchard and Myers, 2010). Interestingly, older drivers are less involved in car-crashes than other motorists. People over the age of 65 make up 18.2% of the French population, but represent only 10.3% of traffic accident victims. Nevertheless, compared to younger drivers, the elderly suffer from more serious bodily injuries that are often fatal. This could be attributed to reduced sensorimotor abilities and more vulnerable bodies^1^.

Elderly people generally exhibit longer RT than younger adults (Hale et al., 1987). There are, however, selective effects associated with task requirements. A difference of about 14% was observed on sensorimotor tasks while larger differences of about 62%, occurred on mental processing tasks (Cerella et al., 1980). Task difficulty is another factor that causes RT to drastically increase although some compensating factors can limit this difference (Hale et al., 1987). Spirduso (1975) showed that elderly people compensated for the effects of aging by regularly practicing a physical activity (e.g., racket sports or handball). Active elderly people and non-active young people exhibited comparable RT during both simple and discrimination RT tasks. Hale et al. (1987) also evidenced that RT in old and young participants were not linear but positively accelerated across a wide variety of non-verbal tasks of increasing complexity (e.g., mental rotation, abstract matching, choice RT or memory scanning). Finally, RT of both old and young people exponentially increased along with task difficulty, but at different rates. Hale et al. (1987) concluded that RT of people aged between 50 and 60 years increased about 10% faster than those of young people, while RT of elderly people (65 to 75 years) increased about 30% faster.

The aim of this experiment was to test whether elderly drivers’ behavior is sensitive to various task loads, as compared to a control group of younger drivers. In particular, our goal was to evaluate the drivers’ strain when confronted with RT tasks similar to those encountered during driving, e.g., requiring an appropriate response to traffic light changes of various difficulty. Our experiment aimed to determine the processes involved in complex tasks such as information processing and decision making while driving. While there is now ample evidence that elderly people’s performances during RT tasks are impaired, it remains unclear whether this conclusion could be applied to more complex and goal-oriented activities. Thus, we tested whether the time required to process traffic-light information and the relevance of decision-making (i.e., response accuracy) were affected as a function of age. We hypothesized that both time to process information and response accuracy would be more impaired for old than for young drivers, due to normal aging (Cantin et al., 2009). We hypothesized that the decrease in performance would thus originate from the normal decrement in cognitive abilities.

## Materials and Methods

### Participants

We selected 25 middle-aged people (from 22 to 44 years, mean being 29.1 with *SD* = 5.5) in the control group, and 31 elderly people. The only inclusion criterion in this group was to be aged over 70 years. The elderly group included participants aged from 70 to 88 years. We formed the groups in a way that both contained an equal repartition of men and women, although the number of women slightly exceeded that of men in the elderly group. All participants reported that they were preferentially right-handed for usual actions although none completed a specific test for laterality (e.g., Edinburg Handedness Inventory). All participants had their driving license since at least 3 years, and had a normal or corrected-to-normal vision. We selected drivers who drove their car regularly, i.e., at least three times per week. The elderly group exhibited the same features as the control group concerning the driving practice. The only difference between the two groups was thus the age.

We endorsed the guidelines of the International Committee of Medical Journal Editors Studies involving human participants. All participants gave written informed consent in accordance with the Declaration of Helsinki. They were aware that they could stop the experiment at any time without giving any information to the experimenters. As the experiment was only based on RT analysis without requiring specific behavior that could be harmful for participants, the Laboratory Council approved the experimental design and did not transmit it to the Ethics Committee which primarily rules on invasive procedures and drug treatments. Elderly participants were recruited by the Renault Company. All participants were not aware of the purposes and expected results of the study. However, they were provided advices regarding their performance (in relation to driving) after the experiment was completed.

### Experimental Design

#### Instructions

**FIGURE 1:**
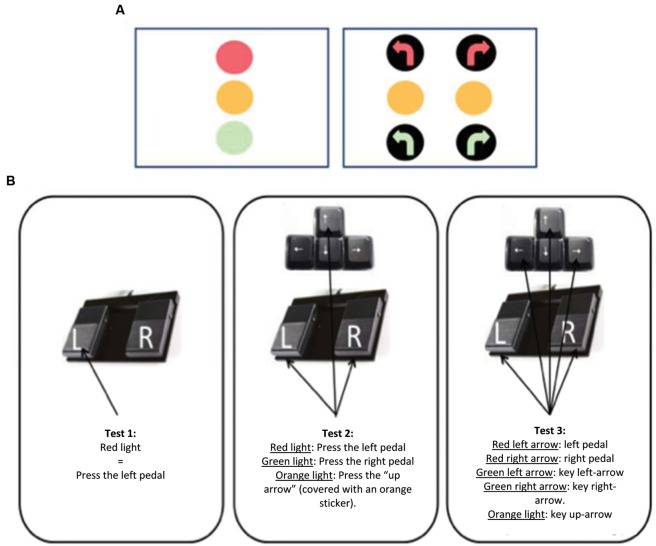
**(A)** Presentation of the traffic lights displayed on the screen. On the left side, lights displayed on the screen during the first and the second tests. On the right side, lights or directional arrows displayed on the screen during the third test. **(B)** Description of each experimental condition (Test 1, Test 2, and Test 3) and the response the participants had to give for each stimulus (light or arrow). Keys that should be pressed on the keyboard were covered with a sticker whose color corresponds to that of the stimulus light.

Each participant completed three different tests on a computer screen displaying different stimuli. The participants were instructed to use either a keyboard or a pedal board to respond to each stimulus, as quickly as possible, within a 2 s time window. This delay is representative of driving situations where quick decisions are required, most being taken within 1.5 s (e.g., decision to brake, see Cantin et al., 2009). We gave specific instructions before each test as follows. For the first test: “*Press the left pedal (as if you had to brake) as early as you see the red light switching on*”. For the second test, the instruction was “*Press the left pedal (as if you had to brake) or the right pedal (as if you had to accelerate) as early as you see the red or the green light switching on, respectively and the “up” arrow of the keyboard when the yellow light switches on*”. For the third test, the participants first learnt the following stimulus-response associations: red right arrow/right pedal, red left arrow/left pedal, green right arrow/right arrow on the keyboard, green left arrow/left arrow on the keyboard and finally yellow right or left arrow/“up” arrow on the keyboard (**Figure 1**). Hence, task requirements were of increasing difficulty from the first to the third test. To sum up, the first task (T1) was a simple RT, whereas the second (T2) and the third (T3) tasks respectively consisted in 3-choice and 5-choice RT. Then, after checking that each stimulus-response association was recalled without any error, the instruction was to press the appropriate pedal (or key) as early as the associated stimulus was triggered. The participants were trained before the experiment started, to make sure that they well understood all instructions – specifically the association between each stimulus and its corresponding key. They were allocated as much time as needed to apply these instructions without errors before starting the experiment.

#### Experimental Stages

Each trial included four stages. First, a black screen was displayed for a random time period from 0.5 to 2.5 s. Second, the traffic light with all lights off was displayed during 1.5 s. Then, one of the lights or arrows lit up during 2 s. Each participant had to respond within this 2 s delay by pressing the appropriate key, otherwise the trial was considered a no-response (i.e., no RT included in the dataset). Finally, mean RT and the cumulative number of incorrect trials, i.e., wrong key or wrong pedal or no-response trials, were displayed during a feedback period of 3s (although we processed wrong responses and no-responses separately). The information display was reset as early as a new test started. To ensure good understanding of instructions, the participants performed the three tests from the easiest (T1) to the most difficult (T3). This progressivity was supposed to facilitate the understanding of the specific instructions attached to each test. Each participant was instructed to respond at best, i.e., achieve the fastest RT associated with the lowest rate of wrong responses. Accordingly, the three tests required a speed/accuracy trade-off with nevertheless priority to accuracy. This fits the requirements of driving scenarios since traffic safety primarily relies on relevant decisions and their associated speed. In the first test, the red light lit 5 times. In the second test, the red, yellow and green lights lit three times each, i.e., a total of 9 trials. In the third test, lights or arrows lit twice each, i.e., a total of 12 trials. Lights or arrows thus lit at random for a total of 26 trials. The whole experiment lasted about 10 min, including rest periods interposed between experimental conditions to prevent mental fatigue. Thus, each trial was separated from each other by about 10 s.

### Behavioral Measurements

The time lapse needed detecting a light or arrow started as early as one of the stimulus was triggered. The rate of wrong responses, i.e., wrong stimulus-response associations, was also considered a dependent variable. Finally, we processed the rate of no-response, i.e., responses provided out of the required time-window or absence of response.

### Data Analysis

All computations were performed using the R statistical software, the *nlme* and *multcomp* packages (R Core Team, 2015)^2^. The *nlme* package can carry out both linear mixed effects models (LMEM) and non-LMEMs. It can easily be linked to the *multcomp* package for multiple comparison. The LMEM (Pinheiro and Bates, 2000) is a statistical tool allowing the combination of numeric covariables with categorical factors (both within-subject and between-subject factors). *Post hoc* comparisons are also easily performed. This explains why we selected *lme* with respect to ANOVA. We successively fitted RT, percentage of wrong responses and percentage no-responses. We used a standard variance-stabilizing transformation for modeling the two percentages (Fisher, 1921). The same LMEM was performed to test the variables described by the following equation:

y(ijk) =M+a(i)+b(j)+ab(ij)+c(ijk)+D(k)+E(ijk)

where i represents conditions (T1, T2, T3), j the middle-aged versus the elderly group and k the participants (*n* = 56). We used M (general mean), and as fixed effects a(i) conditions, within subject, b(j) group, between subject, ab(ij) interaction and c(ijk) numeric covariable, hand-foot ratio. D(k)∼N(0,σS) was the random effects (subject error, N is the normal distribution, 0 is the mean and σS the subjects standard deviation). E(ijk)∼N(0,σ) was the residual error (σ = residual standard deviation). y(ijk) is the dependent variable.

First, the results section provides detailed data from type-II analysis-of-variance (Wald chi-square tests) in **Table 1** according to the principle of marginality (Venables and Ripley, 2002). The level of significance was 5%. Second, we carried out multiple comparisons using the method suitable to mixed effects models from Bretz et al. (2010). The familywise error rate was controlled at level 5%.

**Table 1:** Summary of statistical computations.

Independent variables / Dependent variables	Reaction time (RT)	Response accuracy	No response
Groups	χ^2^_1_ = 166.3, *p* < 0.0001	χ^2^_1_ = 6.5, *p* < 0.01	χ^2^_1_ = 80.0, *p* < 0.0001
Conditions	χ^2^_2_ = 798.0, *p* < 0.0001	χ^2^_2_ = 61.3, *p* < 0.0001	χ^2^_2_ = 108.3, *p* < 0.0001
Groups^∗^Conditions	χ^2^_2_ = 61.3, *p* < 0.0001	χ^2^_2_ = 1.6, *p* = 0.20	χ^2^_2_ = 87.3, *p* < 0.0001
Hand/Foot ratio	χ^2^_1_ = 0.7, *p* = 0.39, NS	χ^2^_1_ = 4.9, *p* = 0.08, MS	χ^2^_1_ = 12.3, *p* < 0.0005
Group effect, T1	*z* = 3.85, *p* < 0.001	*z* = 0.13, *p* = 0.99, NS	*z* = 0.50, *p* = 0.99, NS
Group effect, T2	*z* = 7.00, *p* < 0.001	*z* = 3.16, *p* < 0.01	*z* = 2.83, *p* < 0.03
Group effect, T3	*z* = 13.94, *p* < 0.001	*z* = 1.33, *p* = 0.59, NS	*z* = 12.70, *p* < 0.001
Condition effect, T2-T1 (Young)	*z* = 7.34, *p* < 0.001	*z* = 2.45, *p* = 0.07, MS	*z* = -0.16, *p* = 1, NS
Condition effect, T3-T2 (Young)	*z* = 5.99, *p* < 0.001	*z* = 1.76, *p* < 0.35, NS	*z* = 0.26, *p* = 0.99, NS
Condition effect, T3-T1 (Young)	*z* = 13.42, *p* < 0.001	*z* = 4.24, *p* < 0.001	*z* = 0.10, *p* = 1, NS
Young (T2-T1)-Old (T2-T1)	*z* = 2.42, *p* = 0.09, MS	*z* = 2.20, *p* = 0.15	*z* = 1.68, *p* = 0.39, NS
Young (T3-T2)-Old (T3-T2)	*z* = 2.25, *p* < 0.001	*z* = –1.32, *p* = 0.64	*z* = 7.12, *p* < 0.001
Young (T3-T1)-Old (T3-T1)	*z* = 7.66, *p* < 0.001	*z* = 0.88, *p* < 0.89	*z* = 8.81, *p* < 0.001

**P*-values are indicated after the χ^2^ and the z-values. MS, marginally significant – NS, non-significant.*

## Results

**Table 1** summarizes statistical computations (Wald chi-square tests) through two sets of results. The first set includes groups (middle-aged vs. elderly people), conditions (T1, T2, and T3, as described by **Figure 1**) as independent variables and their interaction. The fourth line details the ratio between hand and foot responses. The second set describes multiple comparisons for each factor independently (group effect, lines 1 to 3). Then, conditions effect is tested by taking into account the differences between T2 and T1, T3 and T2, T3 and T1. Finally, the comparison of middle-aged to elderly people with normalized RTs required to process RT from T2-T1, T3-T1, and T3-T2.

**FIGURE 2:**
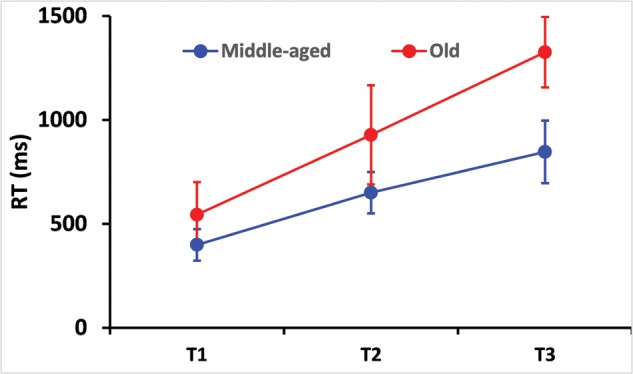
**Reaction time (RT) as a function of independent variables, test difficulty and age.** Obviously, mean RT increased with task difficulty (the experiment consisted of three increasingly difficult tests). Elderly drivers exhibited longer RTs than controls. Difference in RT between young and old people increased with task difficulty. Error bars indicate the standard deviation.

**Figure 2** shows that RT increased with task difficulty from T1 to T3 (χ^2^_2_ = 798, *p* < 0.0001). The elderly group exhibited longer RT than those of the control group (χ^2^_1_ = 166.3, *p* < 0.0001). The first-order interaction Groups*Conditions reached significance (χ^2^_2_ = 61.3, *p* < 0.0001), i.e., the RT difference between the two groups also increased with task difficulty.

Response accuracy decreased as task difficulty increased. Considering both experimental groups, the rates of wrong responses (SD), were 0% (0.0), 7.0% (9.6), and 7.4% (9.2) in the first, second and third test, respectively (χ^2^_2_ = 61.26, *p* < 0.0001). The rates of no-response (SD) were 0.4% (2.7), 4.2% (13.3), and 15.5% (21.0), in the first, second and third test, respectively (χ^2^_2_ = 108.3, *p* < 0.0001).

**FIGURE 3:**
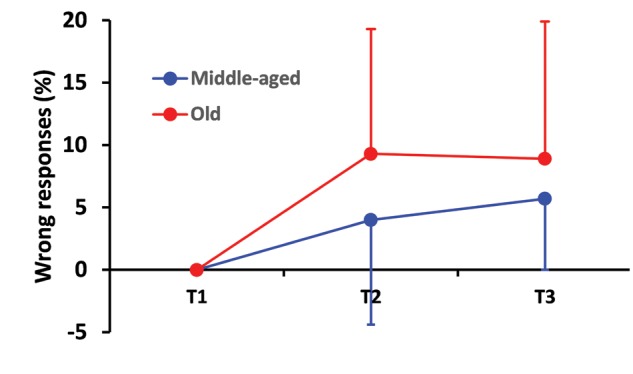
**Percentage wrong responses as a function of test difficulty and age.** This percentage increased more drastically from the first (T1) to the second test (T2) in the elderly group than in the control group. The elderly kept this percentage at the same level while the control group showed a slight increase of this percentage between the second and the third test although young drivers still outperformed those from the elderly group. Error bars indicate the standard deviation.

Elderly participants made more errors than younger people throughout the experiment. The rates of wrong responses were 9.3% (10.0) during T2, 8.9% (11.2) during T3 in the elderly and 4.0% (8.4) during T2, 5.7 (5.7) during T3 for young people (χ^2^_1_ = 6.46, *p* < 0.01). However, the first-order interaction Conditions^∗^Groups did not reach significance (χ^2^_2_ = 4.91, *p* > .05, see **Figure 3**).

**FIGURE 4:**
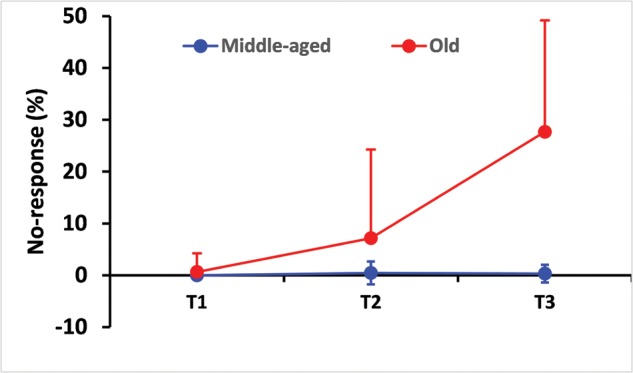
**Percentage of no-responses as a function of test difficulty and age.** Data clearly show that the percentage of no-response increased drastically in the elderly group over the three tests. This means that the elderly group needed much time to process the whole amount of information provided by complex conditions (T2 and T3) where 3-choice RT and 5-choice RT involving the selection of upper or lower limbs to respond adequately. Error bars indicate the standard deviation.

Elderly participants also exhibited higher no-responses rate. The percentages of no-responses were 7.2% (17.3) during T2 and 27.7% (21.4) during T3 in the elderly group, while these were only 0.4% (2.2) and 0.3% (1.7) in the control group (χ^2^_1_ = 80, *p* < 0.0001). The first-order interaction Conditions^∗^Groups reached significance (χ^2^_2_ = 87.3, *p* < 0.0005). Results for the “No-response” condition are displayed in **Figure 4**.

## Discussion

As expected, we observed that RT increased along with task difficulty in both the middle-aged and elderly groups. Similarly, the rate of correct responses decreased while that of no-response increased according to task difficulty. Inter-groups comparisons revealed that the middle-aged group outperformed the elderly group, demonstrating a specific effect of aging impacting both RT and response accuracy (Warshawsky-Livne and Shinar, 2002; Cantin et al., 2009).

Differences in RT always favored the middle-aged group, who reacted faster than the elderly group under the three experimental conditions. The slowing of motor and sensory conduction velocity with aging correlates with some histological changes, e.g., degeneration of horn cells in the spinal cord and neuromuscular junctions (Verdú et al., 2000; Wickremaratchi and Llewelyn, 2006). These neuro-structural changes may particularly account for increased simple RT, where central processing is limited (e.g., during simple RT tasks such as T1). However, between-group differences increased as a function of task difficulty, thus attesting a decrement in brain structure functioning and explaining the slower and less accurate responses.

Driving leads to automated sensorimotor associations between perception and action. The most common link may be between perceiving a visual signal and pressing a pedal. Associating the red light with the left pedal and the green light with the right pedal thus resembled the sensorimotor coupling between traffic lights and brake/acceleration controls during driving (although the left pedal is usually the clutch). Our main results clearly show that this coordination deteriorates in two ways during aging since both RT and response accuracy were impaired in the elderly group. This is consistent with previous studies by Spirduso (1975) and Hale et al. (1987), which showed the prevalence of age-related RT deficit in both simple and choice RT.

Reaction time can be affected by a variety of factors. Therefore, these data should be examined in greater detail. For example, the difference between middle-aged and elderly RT may partly originate from the movement time included in RT measures. Indeed, muscle function is altered in the elderly (Jiménez-Jiménez et al., 2011). However, this may not apply to the present experimental settings since there was no transport phase to push the appropriate key and stop the timer. Feet and hands were placed just above the keys with permanent contact during the entire experiment. A single press stopped the timer, thus limiting the inclusion of movement time as part of RT. This reasoning also applies for the comparison of RT between upper and lower limbs (Simonen et al., 1995). As the limbs were in contact with the keys used to stop the timer, the difference between RTs from the upper and lower limb trials could possibly originate from two other factors. First, neural pathways from the spinal cord to hand muscles are shorter (compared to foot muscles). Since the conduction velocity in the motor pathways and peripheral nerves can reach several tens of meters per second, RT may slightly increase if the response requires distal body segments. Under the conditions of hand and foot contact with the timer, Pfister et al. (2014) reported a significant difference between hand and foot simple RT of about 10 ms, mean data being 318 and 329 ms, respectively. Removing the delay separating foot RT from hand RT can eliminate this difference. The difference can also be neglected in experiments where complex or choice RT are longer than several 100 of milliseconds. Thus, it is less likely that the variance between experimental conditions can be explained by RT differences between hand and foot responses than from the experimental conditions tested. Whenever observed, differences can generally be attributed to the motor selection stage, which is believed to be less challenging for upper limbs than for lower limbs (Kauranen and Vanharanta, 1996; Chan and Chan, 2011; Boisgontier et al., 2014; Pfister et al., 2014). Using a multilimb RT task to investigate the mechanisms of limb selection, Boisgontier et al. (2014) reported that the cerebral operations needed to move the upper limbs required less processing time than for the lower limbs. RT performance depended on the selection of the relevant coordination among the four limbs, and resulted from a weighted combination of recruitment and selection operations. Under these conditions, RT with upper limbs were always faster than those with lower limbs. Mean-difference ranged from 12 to 24 ms and always reached significance. As hypothesized by Boisgontier et al. (2014) differences in RT may account for the ability to process the selection stage. With reference to conduction velocity, a central factor would also account for differences in RT between hand and foot response. Surprisingly, our experiment did not demonstrate any difference between hand RT and foot RT. This probably resulted from the fact that feet responses closely resembled those actually experienced during ordinary car driving. Strong sensorimotor links could have been built between road signs and motor responses with feet. Instead of experimenting with new sensorimotor associations between visual information and foot motor response, the participants may have recalled the perception-action associations built through the daily experience of driving. We nevertheless observed a selective effect of upper and lower limbs on the two other dependent variables. The difference in response accuracy was marginally significant while the rate of no-response reached significance. As the no-response rate corresponded to RT longer than 2000 ms, and was higher in the elderly group, the between-group difference may be age-related.

Overall, the fact that the elderly group exhibited RT differences in all three conditions, with a sharply worsened deficit compared to the other groups, demonstrates that elderly people present alterations during cognitive operations. The different stages of information processing may be slowed by aging, from stimulus perception to central integration with memory retrieval, up to motor response programming (Anstey et al., 2005; Cassavaugh and Kramer, 2009; Ferreira et al., 2012). In other words, the difference in the deficit between middle-aged and elderly people widened when cognitive demand was increased due to complex conditions and/or a limited time allocated to information processing. As task difficulty increased, elderly drivers’ RT increased to a greater extent than that of the middle-aged group. This confirms previous results described by Hale et al. (1987) in a review paper focused on non-verbal RT tasks. The authors reported that RT were predicted by a model of age-related slowing, where task complexity selectively affected information processing of both young and old people. The review by Hale et al. (1987) also emphasized that RT increased exponentially as a function of age but at different rates. In our experiment, whether the relationship between the dependent variables and aging better matches a linear or exponential trend is open to question. However, addressing this issue would require a sample in which age data are continuous and not divided into two categories. The general shape of the slopes nevertheless showed that RT data could fit either linear or exponential trends. When considering the rate of wrong responses, the elderly group’s performance appears drastically impaired when task complexity increases, suggesting that this data could possibly fit an exponential trend better than a linear trend.

Cantin et al. (2009) already observed that elderly drivers exhibited longer RT than younger drivers. In the first test, the elderly group was as accurate as the younger group, thus revealing that the simple task was well performed by both groups, even though elderly drivers needed more time to respond. In the second test (medium difficulty), the elderly group needed more time to respond, made more errors, but exhibited only a slightly higher rate of no-response than the younger group. This means that they probably needed more time to process the information since they more frequently responded outside of the required time-window or did not respond at all. The elderly group exhibited the same profile during the T3 condition, i.e., higher RT than the younger group associated with a drastic increase of no-responses. At first glance, increased error rates would suggest that the elderly group preserved speed at the expense of accuracy. However, the increased no-response rate was also associated with increased RT. Thus, the elderly group’s speed-accuracy profile demonstrated that they had substantial difficulty to process the additional amount of information from T1 to T3. For this reason, they needed more time to complete the task (with increased RT and no-response rates). Several studies mention that elderly people shift the speed-accuracy trade-off toward accuracy. An important issue is raised by Starns and Ratcliff (2010), who suggest that young participants attempt to balance speed and accuracy to achieve correct answers. In contrast, older participants attempt to minimize errors even though their responses are therefore delayed. Van Halewyck et al. (2015) confirmed that older adults favored “play it safe” strategies. This was confirmed in the context of driving (Baldock et al., 2006; Charlton et al., 2006; Blanchard and Myers, 2010) where elderly people showed a tendency to slow down their response time to improve accuracy. The elderly profile in our experiment is in accordance with such strategies. During the most difficult test (the third test), the elderly group exhibited error rates comparable to those of the middle-aged participants, yet they had a higher no-response rate. This result might reveal (i) that the time allocated to process the information (i.e., 2 s) was too short to enable elderly participants to complete the mental operations needed for decision-making or (ii) that the recruitment of both the upper and the lower limbs required information to be processed in parallel, leading to a more complex task. Boisgontier et al. (2014) reported that recruiting several limbs would decrease performance, e.g., increase RT, decrease accuracy, or both. All participants clearly knew the aim of each task and complied with the instructions. Selecting the adequate response among the four limbs increased the complexity of the motor programming stage. Considering that stimuli were easy to perceive and that pressing a key was a simple motor response, decreased performance can be attributed to central operations of response selection.

A way to confirm this assumption is to use a subtractive method to process RT. This consists of normalizing RT by subtracting simple RT values from choice RT values. Boisgontier et al. (2014) suggested that normalized RT conveyed a clearer overview of the factors accounting for RT changes across conditions. Comparing the difference in RT between T2 and T1 in the middle-aged group to those of the elderly refined the initial analysis. Using the same procedure, T3-T2 and T3-T1 differences confirmed that RT discriminated middle-aged from elderly people only in T2 and T3, i.e., the most difficult conditions. We also observed no differences from ‘wrong-responses’ comparisons. The analysis of no-responses thus confirms that the elderly needed more time to process complex information adequately.

Compared to T1, more complex conditions were provided by T2 and T3, which combined 3-choice-RT and 5-choice RT (with upper and lower limbs) respectively, thus eliciting higher temporal constraints. Manipulating speed and the number of choices increased the amount of information to be processed, until the capacities of the elderly people were exceeded. The decline in cognitive capacities resulted in the need for more time to process a larger amount of information, especially when the task required selecting a response with the upper or the lower limbs. This condition increased information processing complexity (Boisgontier et al., 2014). Impairments of cognitive functions resulting from normal aging particularly hamper attention, memory and executive functions (Anstey et al., 2005; Cassavaugh and Kramer, 2009; Ferreira et al., 2012), which are closely involved in operations needed to select (i.e., ignoring irrelevant cues), integrate (i.e., comparing available cues to those memorized during past driving experiences) and evaluate information before selecting the most appropriate response. Elderly participants were able to understand the association between each cue (traffic signal, i.e., lights or arrows) and the corresponding motor response, either on the pedal or on the key. Mental processes bridging the sensory input and the motor output are thus responsible for increasing both the RT and the error rate. The elderly group needed more time to perceive the information, and select the appropriate response, and also exhibited a higher error rate. However, our experiment cannot provide insight into which stages of information processing were specifically impaired.

All information has to be processed in working and long-term memory during the decision-making process. Our study provides evidence that elderly drivers take more time to perform all of these steps. Therefore older drivers are more likely to experience temporal strain under usual driving conditions. This can result in driving errors with potentially serious consequences for the driver’s own safety and that of other road users. Conversely, driving enables elderly motorists to preserve their mobility and autonomy, which is a fundamental aspect of health and well-being (Holahan, 1988; Clarke et al., 2000).

The main implication of our results is to both render elderly drivers more aware of the sensory, motor and cognitive impairments resulting from normal aging, and to encourage them to accept recommendations to help them drive more safely. RT tests, in particular, can be used to make this population more aware of these changes and also make them more readily accepting of safety advice, such as avoiding driving when the traffic is busy, under deteriorated external conditions (e.g., at twilight or night), or reducing speed to allocate more time to select and process relevant information (Baldock et al., 2006; Charlton et al., 2006; Blanchard and Myers, 2010).

## Author Contributions

ES experimental paradigm, experimental sessions, paper writing, and reading. CP experimental paradigm, selection of the participants, paper reading. SC statistical analysis, paper reading. RC providing vehicles and driving infrastructures. FDR statistical advices and paper reading. CC experimental paradigm, experiment supervision, paper writing, and reading.

## Conflict of Interest Statement

The authors declare that the research was conducted in the absence of any commercial or financial relationships that could be construed as a potential conflict of interest.

## Footnotes

1 http://www.preventionroutiere.asso.fr/Nos-publications/Statistiques-d-accidents/Accidents-seniors.

2 https://www.R-project.org
